# Adverse sequelae following revision of a total hip replacement for a fractured ceramic component: case report

**DOI:** 10.1051/sicotj/2015030

**Published:** 2015-10-23

**Authors:** Ling Hong Lee, David Langton, Stephen Green

**Affiliations:** 1 Department of Orthopaedic Surgery, Sunderland Royal Hospital Kayll Road Sunderland SR4 7TP UK; 2 Northern Retrieval Registry, Farndale House, University Hospital North Tees Hardwick Road Stockton-on-Tees TS19 8PE UK

**Keywords:** Ceramic fracture, Ceramic wear, Total hip replacement, Revision

## Abstract

Revision total hip replacement following a fractured ceramic bearing component presents a challenge in the choice of the new bearing implant. A femoral head made of equal or harder material should be implanted to prevent catastrophic wear. Despite this, patients and surgeons must be wary of potential complications.

## Introduction

The choice of bearing material in revision total hip replacement (THR) for fractured ceramic components is debatable although a ceramic head is preferred to metal [1]. In order to prevent excessive wear from the third body ceramic fragments, a material with similar or greater hardness should be used. We report a case of excessive wear of the femoral head after a revision of a fractured ceramic acetabular liner despite maintaining a ceramic coupling.

## Case report

A 63-year-old man presented with an atraumatic undisplaced periprosthetic fracture of the proximal femur 6 weeks after the onset of pain. He initially had difficulty weight bearing but his pain had gradually eased and his mobility had improved by the time of presentation.

He had undergone primary hip replacement 22 years previously, receiving a ceramic on ceramic bearing used in combination with an uncemented femoral stem (Furlong HAC-coated femoral stem, threaded cup, Biolox forte alumina ceramic). Two years after the index procedure, he underwent a revision of the acetabular component and femoral head due to fracture of the ceramic acetabular liner. A ceramic on ceramic articular coupling (HAC-coated CSF cup, Biolox forte alumina ceramic) was used at revision.

Following the revision surgery, the patient remained well, although he had developed a clicking sensation six years before his most recent admission. Medical history includes Crohn’s disease, chronic renal failure and nephrocalcinosis as well as ankylosing spondylitis which required a spinal osteotomy and fusion five years prior to the recent hip complications.

**Figure 1.**
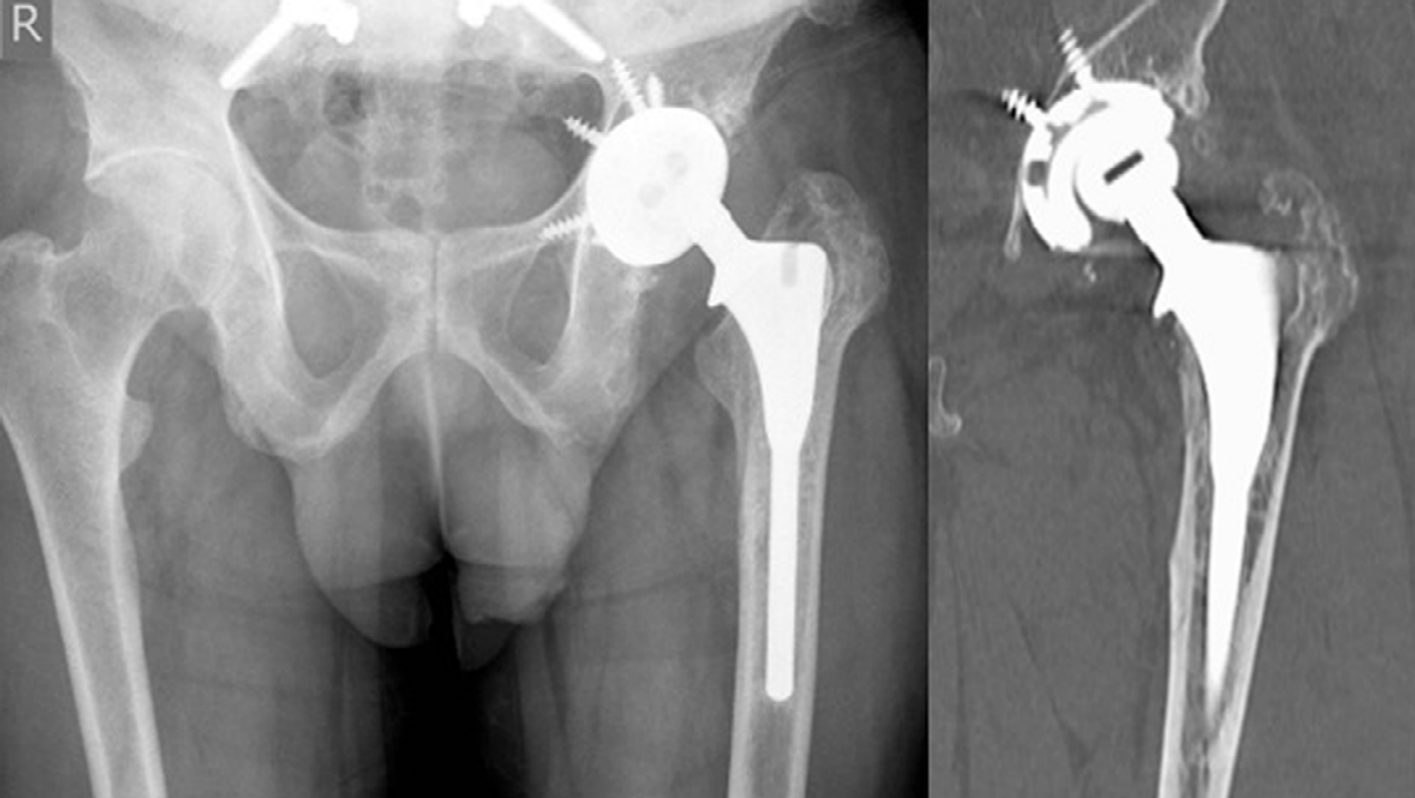
Plain radiograph shows an undisplaced proximal femur periprosthetic fracture involving lesser trochanter. CT shows eccentric wear of the articular surface.

Radiological investigations showed an undisplaced fracture of the lesser trochanter, with evidence of early bony healing. There was obvious eccentric wear of the femoral head (Figure 1). Blood inflammatory markers were unremarkable. Follow-up X-rays showed progressive osteolysis and subsidence of the femoral component. As the patient remained asymptomatic, he declined revision surgery for two years.

### Revision surgery

**Figure 2.**
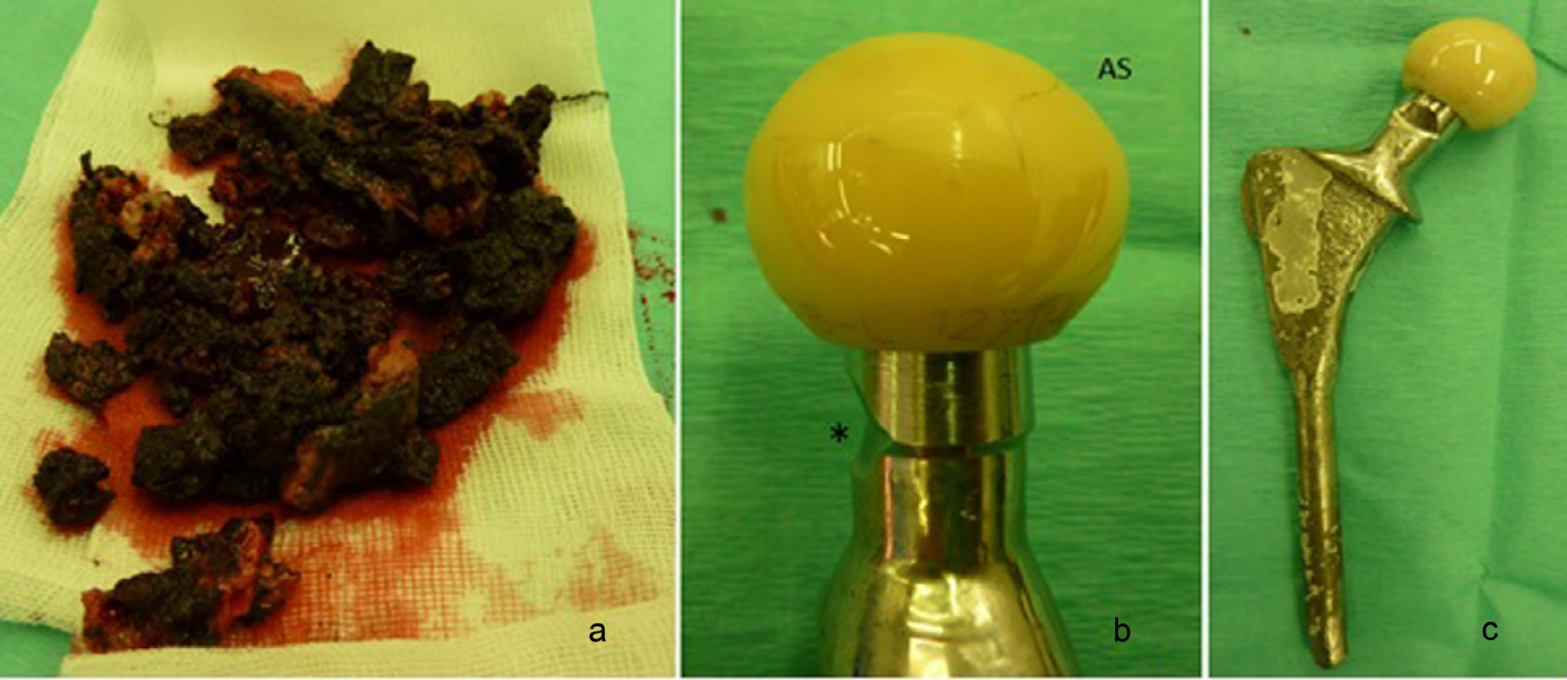
(a) Black metal debris, (b) excessive wear of antero-superior (AS) femoral head and at posterior stem neck (*), (c) femoral stem with attached head.

**Figure 3.**
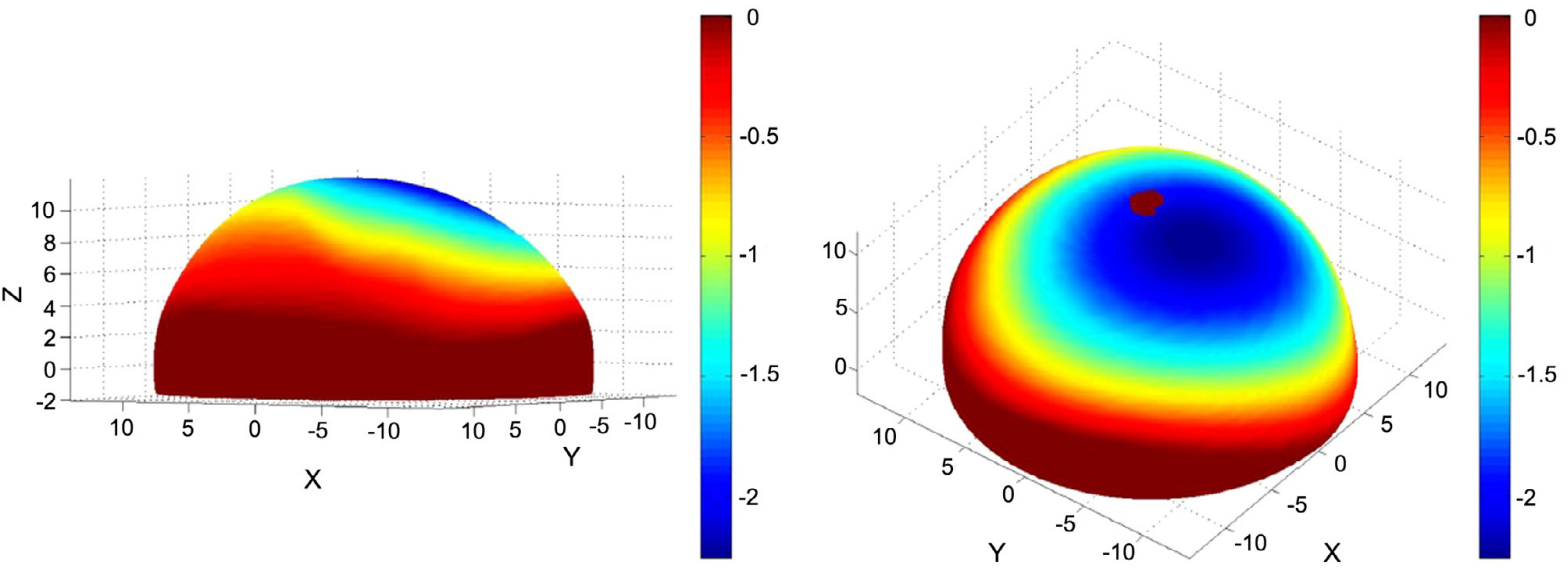
Wear map shows marked asymmetry of the retrieved ceramic femoral head. Red areas indicate unworn surface, blue areas represent maximum wear. Anterior surface to the right. Units in millimetres.

A posterior approach was performed. There was extensive black amorphous metal debris (Figure 2a) and staining of the tissue in the hip and proximal femur. The femoral component was loose due to proximal femoral osteolysis. There was macroscopic eccentric wear at the antero-superior portion of the femoral head (Figures 2b and 3) and significant abrasive surface wear of the femoral stem (Figure 2c). The acetabular liner showed some surface titanium marking but was otherwise macroscopically normal. The cup was well fixed and, as the orientation appeared satisfactory, it was not revised. The stem was revised using a cementless modular femoral implant and ceramic femoral head.

Laboratory analysis of the retrieved ceramic head using validated methodology [2] showed a maximum wear depth of 2.2584 mm (mean linear wear rate of 110 μm/year) and a total volumetric wear of 939.29 mm^3^ (mean volumetric rate of 47 mm^3^/year). There was no significant material loss identified at the stem taper. However, there was massive material loss at the posterior surface of the stem neck consistent with posterior impingement on the cup rim leading to the head subluxing anteriorly (Figure 2b). Accurate assessment of this material loss proved impossible as the wear depths were greater than the coordinate measuring machine probe radius (>2.5 mm), resulting in shanking of the probe during attempts to scan the affected areas.

## Discussion

There is currently no single agreed consensus on the choice of bearing material in revision THR for fractured ceramic components. High rates of failure caused by massive metallosis with stainless steel or cobalt-chrome femoral heads had led to the preference of ceramic head due to its hardness.

This report describes an unusual case of excessive wear of the ceramic femoral head articulating against a ceramic liner. In comparison with volumetric wear rates for ceramic on ceramic bearings reported in the literature, there is a massive difference in the wear magnitude (47 mm^3^/year vs. 0.1–0.2 mm^3^/year) [3]. The ceramic femoral head wear in this patient also far exceeded wear seen in both conventional ultrahigh-molecular-weight and highly-crosslinked polyethylene liners used in hybrid total hip replacement [4]. The reasons may be due to a longer duration of implantation (17 years vs. maximum 11 years) [3, 4] and the indication for revision. The lower wear reported by other authors only involved revision of a primary THR compared to our patient who had already previously undergone revision for fractured ceramic liner. Retained ceramic fragments in the synovium may cause third body wear, but in our patient, although synovectomy had already been performed in the initial revision surgery, there is still a risk of third body wear from retained ceramic fragments.

In this patient, there was abnormal wear from the posterior impingement levering the head anteriorly. Although appearing acceptable to intraoperative examination, the inclination and anteversion were measured to be 59° and 49°, respectively, using the EBRA software (Einzel-Bild-Roentgen-Analysis, University of Innsbruck, Austria). It is also likely that the ceramic femoral head wear was accelerated by third body debris which initially led to a slight reduction in hip offset and instability resulting in subluxation. These factors may explain the patient’s clicking sensation. Although the cause of the periprosthetic fracture is difficult to ascertain, it is very likely to be contributed to the consequence of titanium and ceramic debris from multimodal wear causing osteolysis and bone weakening of this area subjected to high loading stress [5].

Revision surgery for a fractured ceramic bearing component requires meticulous synovectomy to decrease the quantity of ceramic fragments. Implant stability and orientation must be assessed and addressed if required. Surgical technique is also important to prevent early implant failure as is the choice of bearing material. Ceramic on ceramic bearing should be used but the patient and surgeon, must be aware of the potential for future failure.

Patient consent was obtained.

## Conflict of interest

The authors declare that they have no conflict of interest.
